# X-linked intellectual disability type Nascimento is a clinically distinct, probably underdiagnosed entity

**DOI:** 10.1186/1750-1172-8-146

**Published:** 2013-09-21

**Authors:** Johanna Christina Czeschik, Peter Bauer, Karin Buiting, Claudia Dufke, Encarna Guillén-Navarro, Diana S Johnson, Udo Koehler, Vanesa López-González, Hermann-Josef Lüdecke, Alison Male, Deborah Morrogh, Angelika Rieß, Andreas Tzschach, Dagmar Wieczorek, Alma Kuechler

**Affiliations:** 1 Institut für Humangenetik, Universitätsklinikum Essen, Universität Duisburg-Essen, Hufelandstr. 55, 45122, Essen, Germany; 2 Institut für Medizinische Genetik und Angewandte Genomik, Universitätsklinikum Tübingen, Tübingen, Germany; 3 Unidad de Genética Médica, Servicio de Pediatría, Hospital Clínico Universitario Virgen de la Arrixaca, El Palmar, Murcia, Spain; 4 Sheffield Children’s Hospital, Sheffield, UK; 5 MGZ - Medizinisch Genetisches Zentrum, München, Germany; 6 Great Ormond Street Hospital for Children, London, UK

**Keywords:** *UBE2A*, *RAD6A*, *HHR6A*, Ubiquitin-conjugating enzyme, Intellectual disability, Synophrys, Prominent supraorbital ridges, Onychodystrophy

## Abstract

X-linked intellectual disability type Nascimento (MIM #300860), caused by mutations in *UBE2A* (MIM *312180), is characterized by craniofacial dysmorphism (synophrys, prominent supraorbital ridges, deep-set, almond-shaped eyes, depressed nasal bridge, prominent columella, hypoplastic alae nasi, and macrostomia), skin anomalies (hirsutism, myxedematous appearance, onychodystrophy), micropenis, moderate to severe intellectual disability (ID), motor delay, impaired/absent speech, and seizures. Hitherto only five familial point mutations and four different deletions including *UBE2A* have been reported in the literature.

We present eight additional individuals from five families with *UBE2A* associated ID - three males from a consanguineous family, in whom we identified a small deletion of only 7.1 kb encompassing the first three exons of *UBE2A*, two related males with a *UBE2A* missense mutation in exon 4, a patient with a *de novo* nonsense mutation in exon 6, and two sporadic males with larger deletions including *UBE2A*. All affected male individuals share the typical clinical phenotype, all carrier females are unaffected and presented with a completely skewed X inactivation in blood. We conclude that 1.) X-linked intellectual disability type Nascimento is a clinically very distinct entity that might be underdiagnosed to date. 2.) So far, all females carrying a familial *UBE2A* aberration have a completely skewed X inactivation and are clinically unaffected. This should be taken in to account when counselling those families. 3.) The coverage of an array should be checked carefully prior to analysis since not all arrays have a sufficient resolution at specific loci, or alternative quantitative methods should be applied not to miss small deletions.

## Background

Global developmental delay and intellectual disability (ID, IQ < 70) affects up to 3% of the general Western population. X-linked gene defects account for about 10–12% in affected males, and more than 90 X-linked genes causative for ID have been identified [1,2].

X-linked syndromic intellectual disability type Nascimento (MIM #300860) was first described as a distinct entity in 2006 by Nascimento and colleagues [3], who found a nonsense mutation in *UBE2A* (MIM *312180, alternative acronyms *RAD6A*, *HHR6A*) in three intellectually disabled males of a two generation family. Since then, only four further mutations [4,5] and four different deletions [6,7] have been published. The syndrome is characterized clinically by

1.) a pronounced retardation of psychomotor development, i.e. severe impairment or complete lack of speech development, walking ages between 1 and 5 years or no walking ability at all, and seizures in some patients,

2.) a recognisable face (wide face, synophrys, prominent supraorbital ridges, deep-set, almond-shaped eyes, upslanting palpebral fissures, hypertelorism, depressed nasal bridge, prominent columella and hypoplastic alae nasi, macrostomia with downturned corners of the mouth), and a large head circumference or macrocephaly,

3.) skin abnormalities (generalized hirsutism, dry skin, hair whorls, onychodystrophy especially of the feet, and myxedematous appearance), and

4.) urogenital abnormalities (micropenis, cryptorchidism, widely spaced nipples, renal malformations).

Other, less frequently reported associations are malformations of the fingers or toes, heart defects, congenital cataracts, and preauricular pits. White matter abnormalities have been observed in cranial MRI scans [3,6,7].

Here, we describe eight individuals from five families carrying *UBE2A* point mutations or deletions and further delineate the phenotypic spectrum of this entity.

## Materials and methods

### Patients

Eight previously not described patients with intellectual disability type Nascimento were included in this study. Clinical evaluation was carried out at the Departments of Medical Genetics in Essen, Murcia, Tübingen, London and Sheffield. Written informed consent to the study was obtained from the legal representatives of each participant and written consents for publication of the clinical photographs were given. The investigations were performed in accordance with the Declaration of Helsinki protocols.

### DNA extraction

Genomic DNA was extracted from peripheral blood samples using DNA extraction kits and standard protocols (FlexiGene, Qiagen, Hilden, Germany).

### Array CGH analysis

Array CGH analysis in the index patients of family A was performed using a 180 K oligonucleotide array (Cytochip v1.0, BlueGnome, Cambridge, UK). Patients 7 and 8 were analysed using a NimbleGen 135 k WGT CGH microarray with a calculated functional resolution of 0.2 Mb (95% confidence limits). Sample and reference DNAs (peripheral blood) were fluorescently labelled (Cy3-dUTP, Cy5-dUTP) and hybridized according to the manufacturer's protocols (BlueGnome, Cambridge, United Kingdom; NimbleGen Arrays User’s Guide: CGH and CGH/LOH Arrays v9.1, Roche NimbleGen, Madison, WI, USA). Scanning and image acquisition of the Cytochip was performed on an Agilent microarray scanner, scanning of the NimbleGene microarray on an Axon GenePix 4400A Scanner using GenePix Pro 7 software (Molecular Devices, Sunnyvale, CA, USA). Cytochip data analysis was carried out using BlueFuse Multi software (BlueGnome). NimbleGene array raw data was normalized, LOESS correction applied and the data ratios calculated using DEVA v1.01 Software (Roche NimbleGen). The normalized data was processed using Infoquant Fusion v6.0 software (Infoquant, London, UK) with analysis call settings of 3 consecutive probes +/−0.4 Cy3/Cy5 ratio.

Data interpretation was based on the February 2009 human genome sequence assembly (GRCh37/hg19). Conspicuous regions were compared with known CNVs, as provided by the Database of Genomic Variants (http://dgv.tcag.ca/).

### Quantitative real-time PCR (qPCR)

The presence of the *UBE2A* intragenic deletion in family A was investigated by a quantitative real-time PCR assay using the Roche Universal ProbeLibrary System. Part of exon 2 was amplified with primers UBE2A_left: 5'-GTCTGTCTTCCCGAAGGTTG-3' and UBE2A_right: 5'-AATGACCGCGTTCCACAC-3', and detected with the universal probe #19. As an internal control, an assay for the AS-SRO on chromosome 15 was used. The analysis was performed on the LightCycler 480 (Roche). Data were analyzed with the Advanced Relative Quantification method implemented in the LightCycler 480 Software, v1.5.

Real Time quantitative PCR in patients 7 and 8 was carried out using primers (Sigma-Aldrich, St. Louis, USA) from within the *UBE2A* gene. Primers were checked for specificity by melt curve analysis and run on a StepOne Plus Real Time PCR system (Life Technologies Applied Biosystems, California, USA) using the SYBR Green comparative ΔΔCT method. Primers from within genes ACTBL2 and MANEA were used as endogenous control sequence targets. Results were processed using StepOne Plus Software v2.2 with copy number loss or gain indicated by relative quantitation values (RQ).

### Sanger sequencing

For mutation analysis of patient 4, *UBE2A* (ENSG00000077721) coding sequence and adjacent intronic sequences of the longest transcript (ENST00000371558) were taken from ENSEMBL genome browser (http://www.ensembl.org/). Intron-based exon specific primer pairs were designed with Primer3 [8,9]. *UBE2A* exons were amplified using the FastStart Taq DNA Polymerase, dNTPack (Roche) and purified with AmpureXP (Beckman Coulter, Inc) following standard protocols. BigDye Terminator v3.1 Cycle Sequencing Kit was used for sequencing reactions prior to sequencing on a 48-capillary 3730 DNA Analyzer (Applied Biosystems). Sequencing result files were analysed using Megalign (DNASTAR, Inc) and Chromas Version 1.45.

### X-Exome Sequencing

Patient 6 was tested by X-exome analysis, i.e. a next-generation sequencing approach targeted at the coding regions of the X chromosome. In short, a fragmented DNA sample was enriched for the coding and flanking intronic regions of the X chromosome using the Agilent SureSelectXT X-Chromosome in-solution target enrichment kit (Agilent, Santa Clara, CA), and sequencing was performed using the Illumina GAIIx sequencer (2 × 76 paired-end sequencing) (Illumina, San Diego, CA, USA). Putatively pathogenic variants were validated by conventional Sanger sequencing.

We used PolyPhen-2 (http://genetics.bwh.harvard.edu/pph2/), SIFT (http://sift.bii.a-star.edu.sg) and Mutation Taster (http://www.mutationtaster.org/) to predict the possible impact of a missense mutation on the structure and function of the protein.

### X chromosome inactivation analysis

To assess the inactivation status of the X chromosome, DNA methylation of the CAG repeat was studied at the androgen receptor locus (*AR*, Xq12, [10]) and the Fragile-X mental retardation gene locus (*FMR1*, Xq27.3, [11]). Undigested DNA samples and DNA samples digested with the methylation sensitive enzyme HpaII were amplified with fluorescence-tagged PCR primers. A male donor was included as a control for complete HpaII digestion. Fragment length analysis of the PCR products was performed on an ABI 3130XL genetic analyser and the GeneMarker software (Softgenetics, State College, PA, USA). The degree of X inactivation was calculated as (pd1/pu1)/(pd1/pd1 + pd2/pd2), where pd1 and pd2 represent the peak integrals of the stronger and weaker HpaII-digested allele, respectively and pu1 and pu2 are the corresponding peak integrals from the undigested samples.

## Results

### Family A (patients 1–3)

This family presented with two developmentally retarded brothers (patients 1 and 2 in Additional file 1: Figure S1) born to healthy consanguineous parents, and another retarded second degree maternal cousin (patient 3 in Additional file 1: Figure S1). Besides the two affected brothers, there was one more severely developmentally retarded brother, who suffered from L2-hydroxyglutaric-aciduria, which had been excluded in patients 1 and 2.

**Figure 1:**
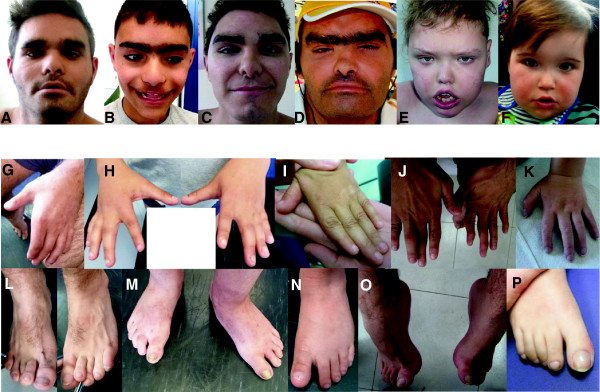
**Clinical photographs of our cohort.** Upper row **(A**-**F)**: Facial phenotypes. Facial phenotype is characterized by a broad face, flat midface, synophrys and/or prominent eyebrows, almond shaped eyes, low nasal bridge, prominent columella of the nose, hypoplastic alae nasi, and macrostomia. A: Patient 1 (family A) at age 19 years, B: Patient 2 (brother of Patient 1, family A) at age 11 years, C: Patient 4 (family B) at age 15 years, D: Patient 5 (family B) at age 38 years, E: Patient 6 at age 7 years, F: Patient 7 at age 5 years. Depending on the ethnic background, synophrys can be only very mild or even absent in individuals with light skin and hair pigmentation (e.g. Patients 6 and 8). Middle row **(G**-**K)** Hands of patients in our cohort with proximally inserted thumbs and short distal phalanges. G: Patient 1. H: Patient 2. I: Patient 3. J: Patient 4. K: Patient 6. Lower row **(A**-**F)** Feet. L: Patient 1 with onychodystrophy especially of the first and fifth toe nail (on the right respectively left hand side). M: Patient 2 with onychodystrophy and sandal gap. N: Patient 3 with sandal gap (status after trauma of the first toe nail). O: Patient 4 with pes cavus. P: Patient 6 with clubbed toe nail.

**Patient 1** was born after 38 gestational weeks by Caesarean section. Birth measurements are unknown. He required phototherapy because of hyperbilirubinaemia. He crawled at the age of 18 months and walked at the age of 3 years. To this day, he had no expressive language. He was reported to have suffered from abscesses of the abdominal skin and perianal region, and from chronic constipation. He was educated in a school for children with special needs and has been living in a children’s home since the age of 12 years because of aggressive behaviour towards his siblings at home. Intermittently, he was reported to exhibit self-mutilating behaviour, which has improved in the past few years. Since the age of 19 years, he has been suffering from generalized seizures. Sleep is often disturbed by waking periods with babbling. A cranial MRI has not been performed. Upon clinical examination at the age of 19 ^11^/_12_ years, he was 1.59 m tall (−2.75 SD), had a weight of 68 kg (BMI 26.9 kg/m^2^) and an OFC of 58 cm (+1.22 SD). He showed a synophrys, tented upper lip, macrostomia, prominent ears (see Figure 1A), proximally inserted thumbs (see Figure 1G), overriding second toes on both feet, and mild onychodystrophy of the feet (see Figure 1L). There seemed to be a rudimentary speech comprehension and ability to follow simple instructions, but no expressive language. Basic communication was facilitated by gestures.

**Patient 2** was born spontaneously after 38 gestational weeks. Birth measurements are unknown. Like his brother, he was treated with phototherapy for hyperbilirubinaemia. He crawled at the age of 18 months and walked at the age of 3 years. He has no expressive language but communicates with gestures. He is educated in a school for children with special needs and is said to have a friendly and helpful temperament. Sleep is light but generally normal. Chronic constipation has been present since birth. A cranial MRI has not been performed. Like his affected brother, he had no formal developmental testing. According to his mother, his mental development is at the stage of a 5-year-old. Hearing was normal, ophthalmological evaluation showed no pathological results except for myopia. A single event resembling a generalized seizure in childhood has been reported. Upon his examination at the age of 11 ^6^/_12_ years, he was 1.46 m tall (−0.2 SD), had a weight of 34 kg (BMI 15.95 kg/m^2^) and an OFC of 52.5 cm (−0.72 SD). He showed a synophrys, tented upper lip, macrostomia (see Figure 1B), prominent ears with a preauricular pit on the right hand side and onychodystrophy of the feet (see Figure 1H and 1M). Two bald spots were present in the occipital region. Lower legs were slightly oedematous. Like his brother, he had rudimentary speech comprehension but no expressive language.

**Patient 3**, the brothers’ second degree cousin, was independently presented as well. He was born after 38 weeks of pregnancy with a birth weight of 3210 g (−0.23 SD) and a length of 50 cm (−0.48 SD). Birth OFC or OFC in early childhood were not recorded. After birth, he exhibited hyperbilirubinaemia, a ventricular septum defect and renal reflux. Developmental delay was evident from the age of 6 months. Starting at an age of 7 months, the patient developed seizures. He was examined at the age of 5 ^9^/_12_ years, when he had a height of 1.08 m (−1.92 SD), a weight of 18 kg (15.43 kg/m^2^) and an OFC of 49 cm (−2.44 SD). He had hypertelorism, a flat nasal bridge, synophrys, proximally inserted thumbs (see Figure 1I), mild onychodystrophy of the feet (Figure 1N) and a lumbar hypertrichosis. He had almost no expressive language (used the words for mother and father), could crawl and walk with support. Cranial MRI showed occipital hyperintense spots that were considered to have been caused by microembolisms leading to focal hypoxia.

**Figure 2:**
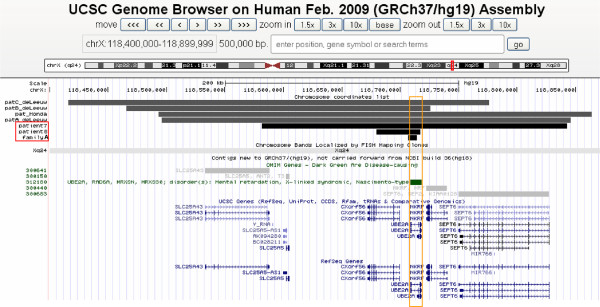
**Comparison of the three Xq24 deletions encompassing *UBE2A *detected in this study (black bars) and the four deletions previously published in the literature (grey bars **[6]**,**[7]**.** The smallest deletion (family A) is just 7.1 kb in size and only affects exons 1–3 of *UBE2A*.

**Figure 3:**
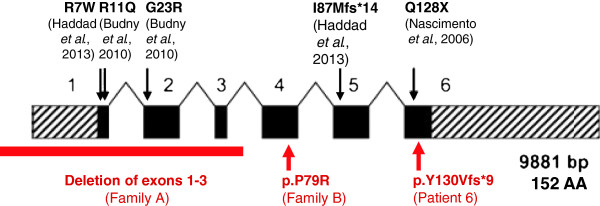
**Scheme of the *UBE2A *** **gene, showing the position of the newly identified (in red, below) and previously published mutations (in black, above; modified after **[4]**).**

Array CGH analysis in patient 2 revealed a small deletion with a minimal size of 7.1 kb encompassing exons 1 to 3 of the *UBE2A* gene (arr[hg19] Xq24(118,679,517x1,118,706,962-118,714,074x0,118,714,378x1)mat, see Figures 2 and 3). The deletion was confirmed by qPCR. The same deletion was diagnosed in his affected brother (patient 1) and in his cousin (patient 3). The clinically unaffected mothers were confirmed to be heterozygous carriers of the deletion. In both females, X inactivation in blood was completely skewed (ratio 100:0).

### Family B (patients 4 and 5)

**Patient 4** (Figure 1C) was the second child of non-consanguineous, Spanish parents. The father had amaurosis on the left side attributed to perinatal factors; mother and sister were healthy. He had a maternal uncle with ID, epilepsy and similar dysmorphic features (**Patient 5**). There was a female first cousin of the maternal grandmother with ID of unknown aetiology. Neither clinical information nor blood was available from her (for pedigree, see Additional file 2: Figure S2).

**Patient 4** was born at 39^+4^ weeks of gestation, after an uneventful pregnancy and delivery, with weight 3750 g (+0.61 SD), length 51 cm (−0.39 SD) and OFC 33 cm (−1.77 SD). APGAR score was 5/8/10. Dysmorphic features and congenital hypothyroidism were apparent at birth. A pineal gland cyst (with no other brain anomalies in the cranial MRI) was surgically removed. The patient had recurrent respiratory and ear infections in childhood, his psychomotor development was delayed (walking at 18 months, marked speech delay). Because of his ID, he attends a special education school. He has no hearing impairment and has not exhibited seizures, regression or autistic behaviour.

Physical examination (Figure 1C, 1J, 1O) at the age of 15 years showed a height of 161 cm (−1.1 SD), a weight of 51.9 kg (BMI 20.02 kg/m^2^) and an OFC of 54 cm (−0.84 SD). He had thick eyebrows, synophrys, upslanting palpebral fissures, light-green iris, a short nose, prominent chin and low-set ears, crowded teeth, highly arched feet, and a bilateral single palmar crease. Genitalia were normal male. Joints were slightly hyperextensible. There was dry skin and generalized hirsutism. Ophthalmologic evaluation showed hyperopia (5 dioptres) and astigmatism (1 dioptre); the skeletal survey showed subluxation of second and third metatarsophalangeal joints of right foot; the abdominal ultrasound and cardiac evaluations had normal results. High resolution karyotype, Fragile-X, subtelomeric MLPA and MLPA of X-linked ID genes (kit MRX106, including *ARX, ARHGEF6, DCX, PQBP1, TM4SF2, IL1RAPL1, RPS6KA3, OPHN1, PAK3, FACL4, GDI1, FMR1, FMR2, SCL6A8;* MRC Holland) and array CGH (Ag 400 k, Qgenomics, Barcelona) had revealed normal results.

**Patient 5** (Uncle of patient 4, Figure 1D) was the fifth child of a healthy and non-consanguineous Spanish couple. It was a diamniotic, dichorionic twin pregnancy. The mother suffered from nausea and vomiting during the whole pregnancy and had a seasonal flu in the second trimester. The patient was the second twin and was born at term by vaginal dystocic delivery. APGAR score was not recorded, but he needed resuscitation. His birth weight was 2800 g (−1.87 SD), birth length and OFC are unknown. Craniofacial dysmorphic features were starkly apparent at birth. He walked at the age of 4 years and showed marked expressive language delay. He had his first seizure when he was 5 years old and was diagnosed with temporal lobe epilepsy. Brain CT scan showed no anomalies. Currently, he communicates with gestures and few words (six to eight) and displays clumsiness and frequent falls. He suffers from chronic constipation. Abdominal ultrasound, cardiac and ophthalmological evaluations revealed no anomalies. There seems to be a hearing impairment although this has not been checked. During the week he lives in a centre for disabled people. On examination at age 38 years, he was 165 cm tall (−1.87 SD), had a weight of 72.7 kg (BMI 26.7 kg/m^2^) and an OFC of 58 cm (+1.22 SD). He presented with a broad face, synophrys, long eyelashes, upslanting palpebral fissures, short nose, macrostomia and down-turned corners of the mouth (see Figure 1D), short and highly arched feet and myxoedematous appearance of the skin.

After being aware of this characteristic phenotype, the diagnosis of X-linked ID type Nascimento was established in these two male patients clinically and Sanger sequencing of *UBE2A* was performed directly. By this, a missense mutation c.236C > G (p.Pro79Arg) in Exon 4 of the *UBE2A* gene was detected. All prediction programs used predicted this substitution to be damaging: PolyPhen-2 predicts this mutation to be probably damaging with a score of 1.000, SIFT as deleterious with a score of 0.00, Mutation Taster as disease-causing. The mutation was confirmed in heterozygous state in the clinically healthy mother, grandmother and one maternal aunt. All informative female carriers had a complete skewing of X inactivation in blood (see Additional file 2: Figure S2).

**Patient 6** (Figures 1E, 1K, 1P) was born at term with normal APGAR values, a length of 55 cm (+1.09 SD), a weight of 4240 g (+1.4 SD) and an OFC of 34 cm (−1.23 SD). Mother and father were aged 38 and 55 years, respectively. For this reason, an amniocentesis was performed and resulted in a normal male karyotype (46,XY). Postnatally, the patient suffered from transient hypoglycaemia, muscular hypotonia and feeding difficulties that required surgical implantation of a percutaneous endoscopic gastrostomy tube (PEG). During his childhood, a global severe retardation with autistic features and central motor coordination defect, as well as epilepsy (tonic-clonic seizures, grand mal seizures and absences) became apparent. In addition, he had a complex cardiac defect: double-outlet right ventricle, inlet ventricular septum defect, mitral stenosis, persistent superior vena cava and pulmonary hypertension, and a hip dysplasia (right > left). Cranial MRI showed a congenital gyration defect in the fronto-opercular region and a tumour of the pinealis of stable size. During his development, progressive global brain atrophy, progressive thickening of the cranial skull base, progressive spasticity of the legs and loss of motor skills were observed. Postnatal karyotype was normal in blood and fibroblasts (46,XY); subtelomere FISH, 6.0 SNP array, and investigation of ATR-X (HbH cells, mutation and deletion analysis) were normal as well. At the age of 7 years, he had a length of 122 cm (−0.32 SD), a weight of 26.1 kg (BMI 17.54 kg/m^2^), and an OFC of 54 cm (+0.98 SD) He was able to vocalize, but had no expressive speech. Crawling and walking few steps with support was possible. Seizures had ceased under valproate therapy. It is assumed that his much more severe clinical course is aggravated by complications of the cardiac defect. He also developed cyanotic lips and finger clubbing.

Patient 6 was included in an exome sequencing study for identification of X-linked genes responsible for developmental delay and ID. A nonsense mutation (c.387Gdup; p.Tyr130Valfs*9) in exon 6 of the *UBE2A* gene was detected. This mutation occurred *de novo*.

**Patient 7** (Figure 1F) was born by emergency section for failure to progress at 38 weeks of gestation. Early pregnancy screening showed raised nuchal thickness. CVS and anomaly scan were normal. His birth weight was 4100 g (+1.81 SD). At, or soon after, birth the patient was diagnosed with talipes and with an inguinal hernia that required surgery; and with laryngomalacia, gastro-oesophageal reflux, ventricular septal defect, patent foramen ovale and patent ductus arteriosus all of which resolved by 2 years of age. He later developed epilepsy, initially presenting with absence attacks at 1 year of age but these later progressed to myoclonic seizures. At the age of 5 years he is able to walk short distances with support. He has about 20 signs for communication and several monosyllables such as “ma” and “da”. He has some self-harming behaviour in the form of head banging and hand biting when frustrated or upset. He is very sensitive to noise. However, he is usually able to communicate his needs; he engages well with people and laughs frequently. He flaps his arms when excited. He also has periods of wakefulness at night when he laughs and chatters to himself. He has persistent neutropenia; respiratory infections occur about twice a year and can be prolonged. MRI showed lack of white matter bulk with delay in maturation of myelination. There was low termination of the spinal cord at L2-L3 with a fatty filum terminale. Examination showed normal growth parameters, central hypotonia, fine hair, a large anterior fontanelle, upslanting palpebral fissures, a large mouth with macroglossia and tented lips (Figure 1F). He had normal fingers and toes.

Array CGH analysis detected a minimal deletion of 261 kb comprising 7 genes/loci including the *UBE2A* gene. The deletion turned out to be maternally inherited (arr[hg19] Xq24(118,564,064x1,118,581,896-118,842,750x0,118,855,888x1)mat; see Figure 2). X inactivation analysis in the mother showed a complete skewing in blood.

**Patient 8** was born at 36 weeks by Caesarean section after failed induction of labour. Birth weight was 3640 g (+1.74 SD). He was tube fed from 1 week until 15 months old. He sat at age 14 months, stood at 18 months and walked with support at 2 ½ years. At this stage he had a few single words. Investigations showed truncus arteriosus, ventricular septum defect, atrial septum defect, patent foramen ovale, and branch pulmonary artery stenosis. Renal scan was normal. He initially had a problem with frequent infections but this has resolved. He has not had a brain MRI. On examination at age 18 months he was 77 cm tall (−2.05 SD) and his OFC was 46.5 cm (−1.56 SD). He had hypertelorism, slightly upslanting palpebral fissures, macrostomia, overriding second toes, lateral deviation of third fingers, small penis, hypospadias, and cryptorchidism.

Array CGH analysis revealed a minimal deletion of 38 kb that comprises only two genes, *UBE2A* and *cXorf56* (arr[hg19] Xq24(118,600,360x1,118,679,518-118,717,453x0,118,717,653x1)mat; see Figure 2). The deletion was inherited from his mother, who showed a completely skewed X inactivation in blood.

## Discussion

In this study, we present eight new patients from five families with X-linked ID type Nascimento. Three individuals of our cohort carry a familial intragenic 7.1 kb deletion of the first three exons of *UBE2A*, two carry a larger deletion encompassing *UBE2A* and additional genes, one carries a *de novo* nonsense mutation and two carry a familial missense mutation.

These five families expand the mutational spectrum underlying this entity, since only five different mutations and four deletions had been described so far. In the original family with X-linked ID type Nascimento [3], Nascimento and co-workers used a candidate gene approach after linkage analysis and detected a nonsense mutation (c.382C > T, p.Gln128*) in *UBE2A*. Although the three index patients of Nascimento and colleagues showed a characteristic clinical phenotype, the next two families were identified only in 2010 by Budny and co-workers [4], who also performed linkage analysis and sequencing of relevant candidate genes in their first family. Their second family was first diagnosed clinically, and *UBE2A* sequencing revealed missense mutations in both families (c.67G > A, p.Gly23Arg; c.32G > A, p.Arg11Gln, respectively [3]). At the same time, a Japanese family with two affected boys [7] and three unrelated affected individuals [6] were published, all detected by array analyses carrying larger overlapping deletions of 240 kb – 370 kb, encompassing the *UBE2A* gene (for clinical characterisation see Table 1, for localisation and affected genes, see Figure 2). Two *UBE2A* mutations were published most recently, a missense and a truncating mutation, identified by X-exome sequencing in two families with two and three affected boys, respectively [5].

**Table 1:** **Clinical description of our patients with ID type Nascimento (OMIM #300860), table modified after de Leeuw et al.**[6]

	**Patient 1**	**Patient 2**	**Patient 3**	**Patient 4**	**Patient 5**	**Patient 6**	**Patient 7**	**Patient 8**
	**(brother of pat. 2)**	**(brother of pat. 1)**	**(cousin of pat. 1&2)**	**(nephew of pat. 5)**	**(uncle of pat. 4)**			
***Gender***	M	M	M	M	M	M	M	M
***Positive family history***	+	+	+	+	+	-	-	-
***Consanguinity***	+	+	-	-	-	-	-	-
***Gestational weeks***	38	38	38	39	40	40	38	36
***Birth weight [g]/[SD]***	n.r.	n.r.	3210/-0.23	3750/+0.61	2800/-1.87	4240/+1.4	4100/+1.81	3640/+1.74
***Birth length [cm]/[SD]***	n.r.	n.r.	50/-0.48	51/-0.39	n.r.	55/+1.09	n.r.	n.r.
***Birth OFC [cm]/[SD]***	n.r.	n.r.	n.r.	33/-1.77	n.r.	34/-1.23	n.r	n.r
***Age at last examination [y]***	19 ^11^/_12_	11 ^6^/_12_	5 ^9^/_12_	15 ^0^/_12_	38	7 ^0^/_12_	2^10^/_12_	6/12
***Height [cm]/[SD]***	159/-2.75	146/-0.2	108/-1.92	161/-1.1	165/-1.89	122/-0.32	98/+0.25	77/-2.05
***Weight [kg]/BMI [kg/m2]***	68/26.9	34/15.95	18/15.43	51.9/20.02	72.7/26.7	26.1/17.54	16.4/17.1	n.r
***OFC [cm]/[SD]***	58/+1.22	52.5/-0.72	49/-2.44	54/-0.84	58/+1.22	54/+0.98	50.3/-0.37	46.5/-1.56
***Development***								
***Crawling***	+ (starting at 18 months)	+ (starting at 18 months)	+	n.r.	n.r.	+	-	n.r
***Walking***	with support (starting at 3 years)	+ (starting at 3 years)	with support	+ (starting at 18 months)	+ (starting at 4 years)	+ (starting at 18 months; progressive spasticity of legs and loss of motor skills)	stands and walks short distances with support	with support at 2.5 years
***Expressive speech***	-	-	few words	+ (first words at 2 years; currently simple sentences)	few words	-	several monosyllables	few at 2.5 years
							20 signs	
							communicates needs	
***Basic speech comprehension***	+	+	+	+	+	+	+	n.r.
***Neurological abnormalities***								
***Seizures***	unspecified (starting at 19 years)	-	complex-focal (starting at 7 months)	-	+ temporal lobe epilepsy (starting at 5 years	tonic-clonic, grand mal, absences (starting at 2 years)	absences.	no
							myoclonic	
***Intellectual disability***	severe	severe	severe	moderate	severe	severe	severe	yes
***White matter abnormalities***	n.r.	n.r.	+	-	-	-	+	n.r
***Hypoplastic cerebellum***	n.r.	n.r.	-	-	-	-	-	n.r
***Other MRI findings***	n.r.	n.r.	n.r.	benign pineal gland cyst	-	congenital gyration defect, pinealis tumour, progressive global brain atrophy	low termination of spinal cord L2-L3	n.r
***Craniofacial dysmorphism***								
***Synophrys***	+	+	+	+	+	+	-	-
***Macrostomia***	+	+	+	+	+	+	+	+
***Short, broad neck***	+	+	+	-	+	+	-	-
***Low posterior hairline***	+	+	+	-	+	n.r.	+	-
***Depressed nasal bridge***	+	+	+	-	+	+	+	+
***Prominent columella and hypoplastic alae nasi***	+	+	+	+	+	+	+	+
***Ocular hypertelorism***	+	+	+	-	-	+	-	+
***Upslanting palpebral fissures***	+	+	-	+	+	-	+	+
***Preauricular pits***	+ (unilateral)	-	-	-	-	-	-	-
***Urogenital abnormalities***								
***Micropenis***	-	+	-	-	-	+	-	+
***Cryptorchidism***	-	-	-	-	-	-	-	+
***Hypospadias***	-	-	-	-	-	-	-	+
***Renal abnormalities***	n.r.	n.r.	vesico-ureteral reflux	-	-	n.r.	n.r.	-
***Skin abnormalities***								
***Generalized hirsutism***	+	+	- (lumbar hypertrichosis)	+	+	-	- (increased hair growthon legs)	-
***Myxedematous appearance***	+	+	-	-	+	+	-	-
***Widely spaced nipples***	+	+	n.r.	-	n.r.	+	+	-
***Dry skin***	+	+	+	+	+		-	
***Hair whorls***	+	+	-	-	-		-	-
***Onychodystrophy***	+	+	-	-	-	-	-	-
***Streaky hyperpigmentation***	-	-	inguinal to thigh (unilateral)	-	-		-	-
***Others***								
***Postnatal hyperbilirubinemia***	+	+	+	-	-	-	-	
***Feet abnormalities***	small, pes cavus	small, pes cavus	-	small, pes cavus; subluxation of second and third metatarsophalangeal joints (right foot)	small, pes cavus	clubbed nail on first toe	bilateral talipes equinovarus	2nd toe overlaps 3rd
***Proximally inserted thumbs***	+	-	+	-	+	+	-	-
***Heart defect***	-	-	ventricular septum defect	-	-	double-outlet right ventricle, inlet ventricular septum defect, mitral stenosis, persistent superior vena cava , pulmonary hypertension	venticular septum defect	truncus arteriosus, ventricular septum defect, atrial septum defect, patent foramen ovale, branch pulmonary artery stenosis
							patent foramen ovale	
							patent ductus arteriosus (resolved by 2 years)	
***Hearing loss***	-	-	-	--	+	-	-	-
***Congenital cataract***	-	-	-	--	-	-	-	-
***Recurrent infections***	recurrent cutaneous abscesses	-	-	recurrent respiratory and ear infections	-	-	prolonged respiratory infections neutropenia	-
***Chronic constipation***	+	+	-	+	+		occasional problems only	-
***Results***								
***UBE2A mutation***				c.236C > G (p.Pro79Arg)	c.236C > G (p.Pro79Arg)	c.387G dup(p.Tyr130Valfs*9)		
***UBE2A deletion***	arr [hg19]Xq24 (118,706,962-118,714,074)x0 mat	arr[hg19] Xq24 (118,706,962-118,714,074)x0 mat	arr[hg19] Xq24 (118,706,962-118,714,074)x0 mat				arr[hg19] Xq24 (118,581,896-118,842,750)x0 mat	arr[hg19] Xq24 (118,679,518-118,717,453)x0 mat

n.r. = not recorded.

On the basis of this still very limited number of cases published in the last seven years, ID type Nascimento seems to be a quite rare condition, and *UBE2A* deletions and mutations seem to occur with an equal frequency. These proportions might change with the broader use of next generation sequencing technologies like X-exome analysis that will lead to the detection of more underlying mutations in individuals with ID and also of smaller, intragenic deletions.

The detection of this small intragenic deletion of only 7.1 kb in our family 1 (see Figure 2) by the Cytochip v1 180 K array was quite fortunate since this size is on the one hand below the evaluation threshold of most array protocols. On the other hand, several array types do not even cover this locus with sufficient markers (see Additional file 3: Figure S3). The deletion would have been missed, for instance, by the Affymetrix arrays. Therefore, if a specific clinical diagnosis is suspected, the coverage of an array should be checked carefully prior to a planned analysis, or alternative quantitative methods should be applied in order not to miss small deletions.

Although almost all individuals with ID type Nascimento were identified first by molecular methods, we assume that this condition is clinically recognisable. The characteristic *UBE2A* phenotype, consistently present in all individuals in the literature [3-7] as well as in our cohort (see Table 1) includes intellectual disability (mostly moderate to severe) with no or almost no expressive speech, limited walking abilities and a recognisable facial gestalt. The facial phenotype is characterized by a broad face, flat midface, almond shaped eyes, ocular hypertelorism, low nasal bridge, prominent columella of the nose, hypoplastic alae nasi, and macrostomia. Depending on the ethnic background, a synophrys can be very mild or even absent in individuals with light hair and skin pigmentation (see Patients 6 and 8), but still, the eyebrows appear straight, the supraorbital ridges prominent. Therefore even in patients without the pronounced synophrys the eye region remains characteristic for this entity.

Features that are variably present are seizures in almost 2/3 of the patients (62%), abnormalities in the cranial MRI in about half of the patients (47%, esp. white matter abnormalities), genital or renal abnormalities (e.g. micropenis, ureteral reflux) in about 2/3 of the patients (in 65%,), hirsutism or other abnormalities of the skin (e.g., myxedematous appearance, widely spaced nipples, dry skin, hair whorls and onychodystrophy, especially of the feet in up to 3/4 of the patients), heart defects, and less frequently various digital anomalies (proximally inserted thumbs, short thumbs, short fingers, slender fingers) (see Table 2). According to the original publication [3], the onset of onychodystrophy is in or after puberty, which is consistent with the findings in our patients. As in many syndromes, the phenotype changes with age, and especially hair and skin pattern become more pronounced in elder *UBE2A* patients. In one of our patients (patient 3), streaky hyper-/hypopigmentation was identified in the inguinal region, leading to the initial clinical diagnosis of “Ito syndrome”. Hypopigmented spots were also described in two of Budny’s patients [4]. Postnatal hyperbilirubinaemia and severe chronic constipation were present in patients from one family, possibly as a coincidental finding. If both signs are present more frequently in X-linked ID type Nascimento than in the general population can only be determined by evaluation of larger patient cohorts. Hearing loss and congenital cataract were previously described in two patients with deletions [6], but were not present in any other patients. These findings could also be due to other genes involved in the deletion or just have occurred coincidentally.

**Table 2:** Summary of clinical findings in our and the previously published families with Intellectual Disability (ID) type Nascimento (OMIM #300860)

	**Nascimento et al.**	**Budny et al. (Fam 1)**	**Budny et al. (Fam 2)**	**Honda et al.**	**De Leeuw et al. (Pat A)**	**De Leeuw et al. (Pat B)**	**De Leeuw et al. (Pat C)**	**Haddad et al. (Fam 1)**	**Haddad et al. (Fam 2)**	**Family A (Pat 1–3)**	**Family B (Pat 4–5)**	**Pat 6**	**Pat 7**	**Pat 8**	**in total**
**Mutation**	Truncating	Missense	Missense	Deletion	Deletion	Deletion	Deletion	Truncating	Missense	Deletion	Missense	Truncating	Deletion	Deletion	7 Deletions
	p.Q128X	p.G23R	p.R11Q	(370 kb)	(350 kb)	(240 kb)	(360 kb)	p.I87Mfs*14	p.R7W	(7.1 kb)	p.P79R	p.Y130Vfs*9	(261 kb)	(38 kb)	7 Mutations
**No[a]**	3	4	1	2	1	1	1	2	3	3	2	1	1	1	26
**Skewed X-inactivation in carriers**	yes	yes	n.r.	yes	yes	yes	yes	n.r.	yes	yes	yes	*de novo*mutation	yes	yes	in all female carriers investigated
**Intellectual disability**	3/3	4/4	1/1	2/2	1/1e	1/1	1/1	2/2	3/3	3/3	2/2	1/1	1/1	1/1	26/26 (100%)
**Motor delay**	3/3	4/4	1/1	2/2	1/1	1/1	1/1	2/2	3/3	3/3	2/2	1/1	1/1	1/1	26/26 (100%)
**Speech impairment**	3/3	4/4	1/1	2/2	1/1	1/1	1/1	2/2	3/3	3/3	2/2	1/1	1/1	(1/1)	26/26 (100%)
**Facial dysmorphism**	3/3	4/4	1/1	2/2	1/1	1/1	1/1	2/2	(3/3)	3/3	2/2	1/1	1/1	1/1	26/26 (100%)
**Skin changes**	3/3	3/4	1/1	2/2	1/1	0/1	1/1	1/2	2/3	3/3	2/2	1/1	0/1	0/1	20/26 (77%)
**Hirsutism**	3/3	3/4	1/1	2/2	0/1	0/1	1/1	2/2	0/3	2/3	2/2	0/1	(1/1)	0/1	17/26 (65%)
**Urogenital anomalies**	3/3	3/4	1/1	2/2	1/1	1/1	1/1	0/2	2/3	1/3	0/2.	1/1	0/1	1/1	17/26 (65%)
**Seizures**	3/3	3/4	0/1	2/2	1/1	1/1	1/1	0/2	0/3	2/3	1/2	1/1	1/1	0/1	16/26 (62%)
**Behavioural abnormalities**	n.r.	2/2	n.r.	n.r.	n.r.	n.r.	n.r.	1/2	2/3	1/3	0/2	1/1	1/1	n.r.	8/14 (57%)
**White matter abnormalities**	2/2	0/2	n.r.	2/2	1/1	1/1	0/1	0/1	1/2	0/1*	0/2	0/1*	1/1	n.e.	8/17 (47%)
**Heart defect**	0/3	0/4	0/1	2/2	1/1	1/1	1/1	1/2	0/3	1/3	0/2	1/1	1/1	1/1	10/26 (38%)

[a] number of affected individuals with available clinical data.

n.r. not recorded, n.e. not examined.

*see text.

Neutropenia and frequent respiratory infections occurred in our patient 7. Also patient 4 suffered from frequent respiratory and ear infections and had neutrophil levels in the range of low normal to mild neutropenic. Neutrophils were normal in patient 2, 3 and 8 (who initially also had problems with infections), data for patient 1, 5 and 6 were not available. In the literature, patient C described by de Leeuw [6] was also found to be neutropenic, with hypogammaglobulinemia and a low B cell count. Recurrent infections also occurred in patient A of this publication but without detectable immunodeficiency [6]. Nothing is reported on frequent infections or neutropenia in the other publications [3-7]. Therefore, the number of cases is too small to draw a conclusion as to whether neutropenia is an associated finding or occurred coincidentally.

A search in the DECIPHER database ([12], status as of June 18, 2013) revealed two patients with overlapping deletions including *UBE2A*, case 274833 with features of the autistic spectrum and delayed speech carrying a 280 kb deletion including 5 genes, and case 272966 without documented phenotypic features with a 60 kb deletion including 3 genes and an additional small 1q duplication. To our knowledge there is no description in the literature of males with larger deletions containing Xq24 and especially *UBE2A*, presumably because larger aberrations would not have been viable in a male karyotype constellation.

Previously, a causal role of neighbouring genes of *UBE2A*, such as *SLC25A5*, in the genesis of heart defects in patients with *UBE2A*-encompassing large deletions has been hypothesized [6,7]. However, in our cohort, heart defects were not only present in the deletion patients 7 and 8, but also in two patients with intragenic aberrations (patient 3 with an intragenic deletion, and patient 6 with a nonsense mutation). This suggests that heart defects might be a variable feature of X-linked ID type Nascimento, and that a *UBE2A* defect is sufficient for the development of this sign or is a coincidental finding.

In our cohort, X inactivation was completely skewed (about 100:0) in blood of all female carriers of *UBE2A* aberrations. All female carriers were clinically unaffected. This is in accordance with the published results, where all proven [3-6] or presumptive obligatory female carriers [7] in whom this analysis was performed had a skewed X inactivation (see also Table 2). No affected female individuals are known so far. One can speculate that a clinical phenotype could occur in female carriers with random X inactivation.

A similar effect of the X inactivation pattern on phenotypic expression is known in female carriers of a *MECP2* duplication. The majority of female *MECP2* duplication carriers are unaffected due to a preferred inactivation of the aberrant X-chromosome. Random X inactivation was found to cause an associated phenotype in females that is distinct from those in males but can be as severe [13,14].

Therefore, also in genetic counseling of females with *UBE2A* deletions or mutations (especially in prenatal diagnosis), the X inactivation status should be investigated and in case it is random, possible associated phenotypic features have to be discussed.

*UBE2A* (also known as *HHR6A* or RAD6A, OMIM *312180) encodes for an ubiquitin conjugating enzyme (E2). E2 enzymes are together with ubiquitin-activating enzymes (E1) and ubiquitin ligases (E3) involved in the ubiquitination process [15].

Until recently, it was not known, how *UBE2A* or mutations in it exactly affect neuronal function. Very recently, Haddad and co-workers identified RAD6A as a regulator of Parkin-dependent mitophagy and established a critical role for RAD6A in maintaining neuronal function [5]. They could show that drosophila deficient for *dRad6* display defective synaptic function as a consequence of mitochondrial failure. They also investigated mouse *mRad6a* (Ube2a) knockout and patient derived hRad6a (Ube2a) mutant cells and could show that RAD6A as an E2 ubiquitin conjugating enzyme interacts with an E3 ubiquitin ligase such as Parkin resulting in ubiquitination of mitochondrial proteins to facilitate the clearance of dysfunctional mitochondria in cells. The authors postulate that maintaining a healthy mitochondrial pool in vivo is critical to maintain normal synaptic transmission, which is potentially an important element involved in the aetiology of ID.

Since ID type Nascimento is a clinically recognizable entity, some differential diagnoses with overlapping phenotypes have to be considered. There are mainly two also X-linked conditions that have to be discussed: in Cabezas syndrome (MIM #300354), intellectual disability, severe speech impairment, gait abnormalities, abnormal hair whorls, small feet, small male genitalia, hypospadias and cryptorchidism are characteristic features [16]. Mutations in *CUL4B* have been identified as underlying cause [17]. Börjeson-Forssman-Lehmann syndrome (BFLS, MIM #301900), caused by mutations or deletions in *PHF6*[18] shares facial characteristics with X-linked ID type Nascimento (especially prominent supraorbital ridges, prominent columella and hypoplastic alae nasi and coarsening of facial features during adolescence) as well as intellectual disability and small external genitalia in boys. However, intellectual disability is usually described as mild to moderate in BFLS patients, in contrast to a moderate to severe degree of intellectual disability in X-linked ID type Nascimento. Other features of BFLS not usually present in patients with X-linked ID type Nascimento are truncal obesity, gynecomastia, tapering fingers and fleshy ear lobes.

## Conclusion

The distinct pattern of facial dysmorphism and other physical and developmental characteristics of patients with X-linked ID type Nascimento suggests that this is a clinically recognizable entity that might be underdiagnosed to date. So far, all females carrying a familial *UBE2A* aberration have a completely skewed X inactivation and are clinically unaffected. This should be taken in to account when counselling those families. If this condition is clinically suspected, sequence analysis plus dosage analysis should be undertaken. The coverage of an array should be checked carefully prior to an analysis since not all arrays have a sufficient resolution at this locus, or alternative quantitative methods like qPCR or MLPA should be applied not to miss small deletions.

## Competing interests

The authors declare that they have no competing interests.

## Authors’ contributions

JCC, AK, and DW were involved in design, acquisition and analysis of data, and drafting of the manuscript. VLG, EGN, DSJ, AM, and AR were involved in acquisition and analysis of clinical data and made contributions to the draft of the manuscript. PB, KB, CD, UK, H-JL, DM, and AT were involved in acquisition and analysis of molecular data and made contributions to the draft of the manuscript. All authors read and approved the final manuscript.

## Supplementary Material

Additional file 1: Figure S1 Pedigree of Family A with three affected individuals (patients 1, 2, 3) with molecularly proven *UBE2A* deletion and several individuals showing clinical signs suggestive of X-linked ID type Nascimento based on photos/history (molecular proof was not possible so far). Click here for file

Additional file 2: Figure S2 Pedigree and results of X inactivation study and mutation analysis (electropherograms) in Family B with two affected male individuals (patients 4 and 5). All three female carriers are healthy. Click here for file

Additional file 3: Figure S3 Coverage of the *UBE2A* region with array markers – comparison of different commercially available array types (according to the UCSC browser, hg19, as of June 2013). Not all array types sufficiently cover the *UBE2A* region. Two arrays depicted here (Affymetrix SNP 6.0 and Affymetrix Cytoscan HD array) contain no markers at all or only markers in the last exon so that intragenic deletions as in family 1 would have been missed. Click here for file

## References

[B1] Ropers HH Genetics of early onset cognitive impairment Annu Rev Genomics Hum Genet 2010 8 161 187 10.1146/annurev-genom-082509-141640 20822471

[B2] Flore LA Milunsky JM Updates in the genetic evaluation of the child with global developmental delay or intellectual disability Semin Pediatr Neurol 2012 8 173 180 10.1016/j.spen.2012.09.004 23245550

[B3] Nascimento RMP Otto PA de Brouwer APM Vianna-Morgante AM UBE2A, which encodes a ubiquitin-conjugating enzyme, is mutated in a novel X-linked mental retardation syndrome Am J Hum Genet 2006 8 549 10.1086/507047 16909393 PMC1559544

[B4] Budny B Badura-Stronka M Materna-Kiryluk A Tzschach A Raynaud M Latos-Bielenska A Ropers HH Novel missense mutations in the ubiquitination-related gene UBE2A cause a recognizable X-linked mental retardation syndrome Clin Genet 2010 8 541 10.1111/j.1399-0004.2010.01429.x 20412111

[B5] Haddad DM Vilain S Vos M Esposito G Matta S Kalscheuer VM Craessaerts K Leyssen M Nascimento RM Vianna-Morgante AM Mutations in the intellectual disability gene ube2a cause neuronal dysfunction and impair parkin-dependent mitophagy Mol Cell 2013 8 831 843 10.1016/j.molcel.2013.04.012 23685073

[B6] de Leeuw N Bulk S Green A Jaeckle-Santos L Baker LA Zinn AR Kleefstra T van der Smagt JJ Vianne Morgante AM de Vries BBA UBE2A deficiency syndrome: Mild to severe intellectual disability accompanied by seizures, absent speech, urogenital, and skin anomalies in male patients Am J Med Genet A 2010 8 3084 10.1002/ajmg.a.33743 21108393

[B7] Honda S Orii KO Kobayashi J Hayashi S Imamura A Imoto I Nakagawa E Goto Y-i Inazawa J Novel deletion at Xq24 including the UBE2A gene in a patient with X-linked mental retardation J Hum Genet 2010 8 244 10.1038/jhg.2010.14 20339384

[B8] Koressaar T Remm M Enhancements and modifications of primer design program Primer3 Bioinformatics 2007 8 1289 1291 10.1093/bioinformatics/btm091 17379693

[B9] Untergasser A Cutcutache I Koressaar T Ye J Faircloth BC Remm M Rozen SG Primer3–new capabilities and interfaces Nucleic Acids Res 2012 8 e115 10.1093/nar/gks596 22730293 PMC3424584

[B10] Sharp A Robinson D Jacobs P Age- and tissue-specific variation of X chromosome inactivation ratios in normal women Hum Genet 2000 8 343 349 10.1007/s004390000382 11129333

[B11] Carrel L Willard HF An assay for X inactivation based on differential methylation at the fragile X locus, FMR1 Am J Med Genet 1996 8 27 30 10.1002/(SICI)1096-8628(19960712)64:1<27::AID-AJMG3>3.0.CO;2-O 8826444

[B12] Firth HV Richards SM Bevan AP Clayton S Corpas M Rajan D Van Vooren S Moreau Y Pettett RM Carter NP DECIPHER: Database of Chromosomal Imbalance and Phenotype in Humans Using Ensembl Resources Am J Hum Genet 2009 8 524 533 10.1016/j.ajhg.2009.03.010 19344873 PMC2667985

[B13] Grasshoff U Bonin M Goehring I Ekici A Dufke A Cremer K Wagner N Rossier E Jauch A Walter M De novo MECP2 duplication in two females with random X-inactivation and moderate mental retardation Eur J Hum Genet 2011 8 507 512 10.1038/ejhg.2010.226 21326285 PMC3083613

[B14] Bijlsma EK Collins A Papa FT Tejada MI Wheeler P Peeters EA Gijsbers AC van de Kamp JM Kriek M Losekoot M Xq28 duplications including MECP2 in five females: Expanding the phenotype to severe mental retardation Eur J Med Genet 2012 8 404 413 10.1016/j.ejmg.2012.02.009 22522176 PMC3383992

[B15] Bhat KP Greer SF Proteolytic and non-proteolytic roles of ubiquitin and the ubiquitin proteasome system in transcriptional regulation Biochim Biophys Acta 2011 8 150 10.1016/j.bbagrm.2010.11.006 21184853

[B16] Cabezas DA Slaugh R Abidi F Arena JF Stevenson RE Schwartz CE Lubs HA A new X linked mental retardation (XLMR) syndrome with short stature, small testes, muscle wasting, and tremor localises to Xq24-q25 J Med Genet 2000 8 663 668 10.1136/jmg.37.9.663 10978355 PMC1734699

[B17] Tarpey PS Raymond FL O'Meara S Edkins S Teague J Butler A Dicks E Stevens C Tofts C Avis T Barthorpe S Buck G Cole J Gray K Halliday K Harrison R Hills K Jenkinson A Jones D Menzies A Mironenko T Perry J Raine K Richardson D Shepherd R Small A Varian J West S Widaa S Mallya U Mutations in CUL4B, which encodes a ubiquitin E3 ligase subunit, cause an X-linked mental retardation syndrome associated with aggressive outbursts, seizures, relative macrocephaly, central obesity, hypogonadism, pes cavus, and tremor Am J Hum Genet 2007 8 345 10.1086/511134 17236139 PMC1785336

[B18] Gécz J Turner G Nelson J Partington M The Börjeson-Forssman-Lehman syndrome (BFLS, MIM #301900) Eur J Hum Genet 2006 8 1233 1237 10.1038/sj.ejhg.5201639 16912705

