# Supplementary material for: X-linked intellectual disability type Nascimento is a clinically distinct, probably underdiagnosed entity
Source: Orphanet J Rare Dis. 2013 Sep 21;8:146. doi: 10.1186/1750-1172-8-146 (PMC4015352; doi:10.1186/1750-1172-8-146)
Supplement: Additional file 1: Figure S1 — Pedigree of Family A with three affected individuals (patients 1, 2, 3) with molecularly proven UBE2A deletion and several individuals showing clinical signs suggestive of X-linked ID type Nascimento based on photos/history (molecular proof was not possible so far). [file 1750-1172-8-146-S1.pptx]

## Slide 1
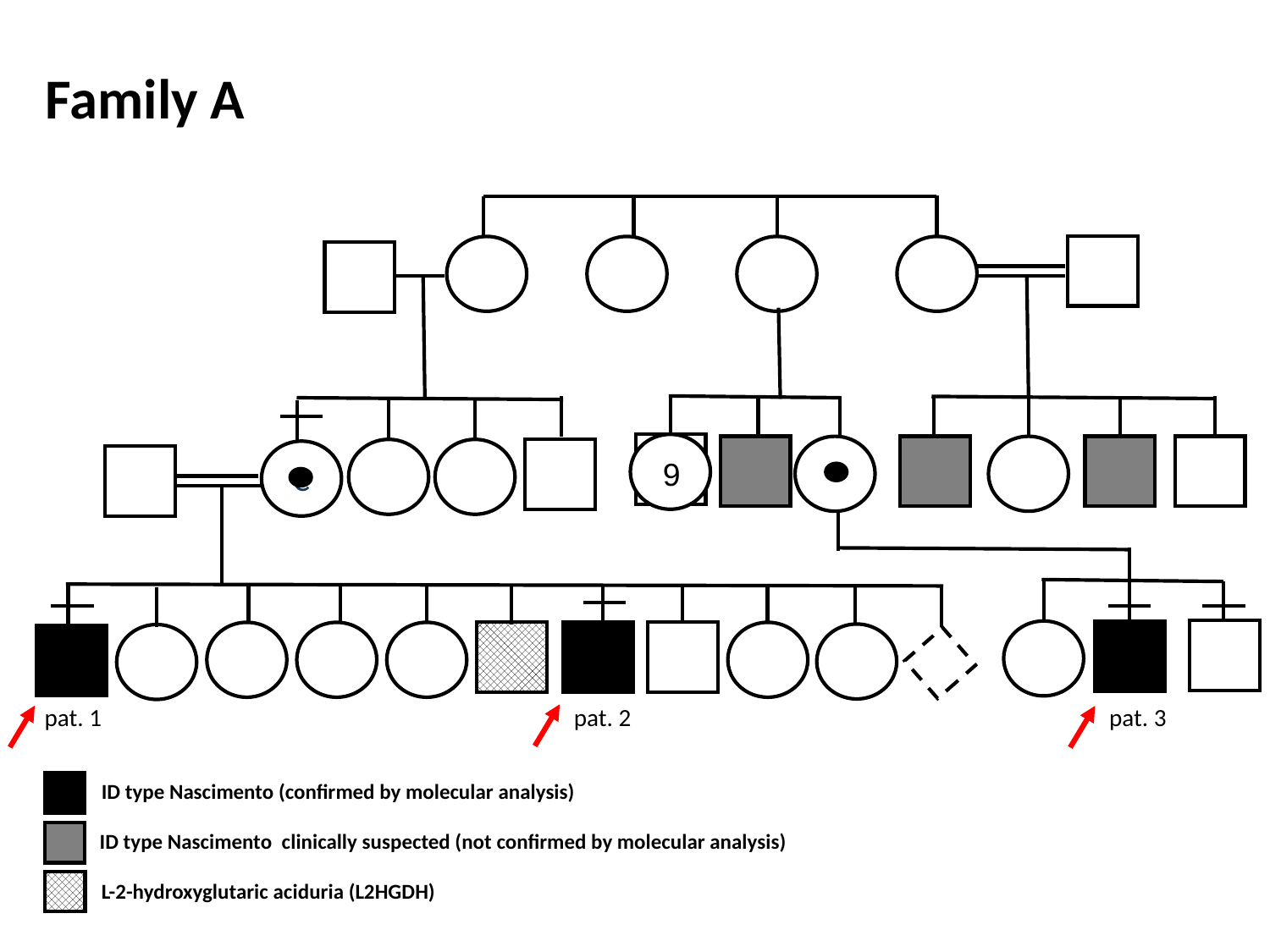

Family A
C
9
pat. 1
pat. 2
pat. 3
ID type Nascimento (confirmed by molecular analysis)
ID type Nascimento clinically suspected (not confirmed by molecular analysis)
L-2-hydroxyglutaric aciduria (L2HGDH)
