# Supplementary material for: X-linked intellectual disability type Nascimento is a clinically distinct, probably underdiagnosed entity
Source: Orphanet J Rare Dis. 2013 Sep 21;8:146. doi: 10.1186/1750-1172-8-146 (PMC4015352; doi:10.1186/1750-1172-8-146)
Supplement: Additional file 2: Figure S2 — Pedigree and results of X inactivation study and mutation analysis (electropherograms) in Family B with two affected male individuals (patients 4 and 5). All three female carriers are healthy. [file 1750-1172-8-146-S2.ppt]

## Slide 1
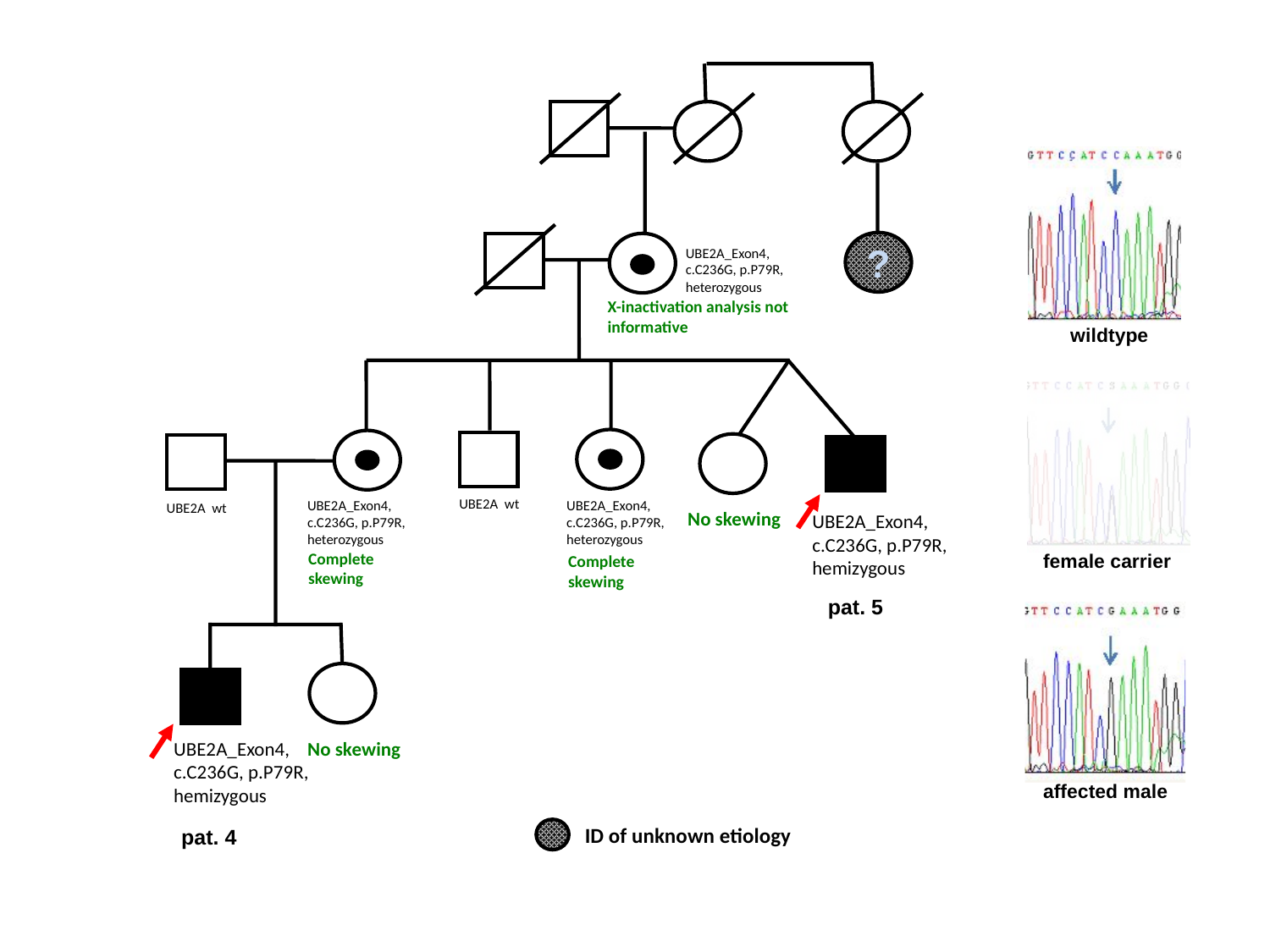

?
UBE2A_Exon4,
c.C236G, p.P79R, heterozygous
X-inactivation analysis not informative
wildtype
UBE2A wt
UBE2A_Exon4,
c.C236G, p.P79R, heterozygous
UBE2A_Exon4,
c.C236G, p.P79R, heterozygous
UBE2A wt
No skewing
UBE2A_Exon4,
c.C236G, p.P79R, hemizygous
Complete skewing
female carrier
Complete skewing
pat. 5
No skewing
UBE2A_Exon4,
c.C236G, p.P79R, hemizygous
affected male
ID of unknown etiology
pat. 4
