# Supplementary figures and images for: X-linked intellectual disability type Nascimento is a clinically distinct, probably underdiagnosed entity
Source: Orphanet J Rare Dis. 2013 Sep 21;8:146. doi: 10.1186/1750-1172-8-146 (PMC4015352; doi:10.1186/1750-1172-8-146)

## Slide 1
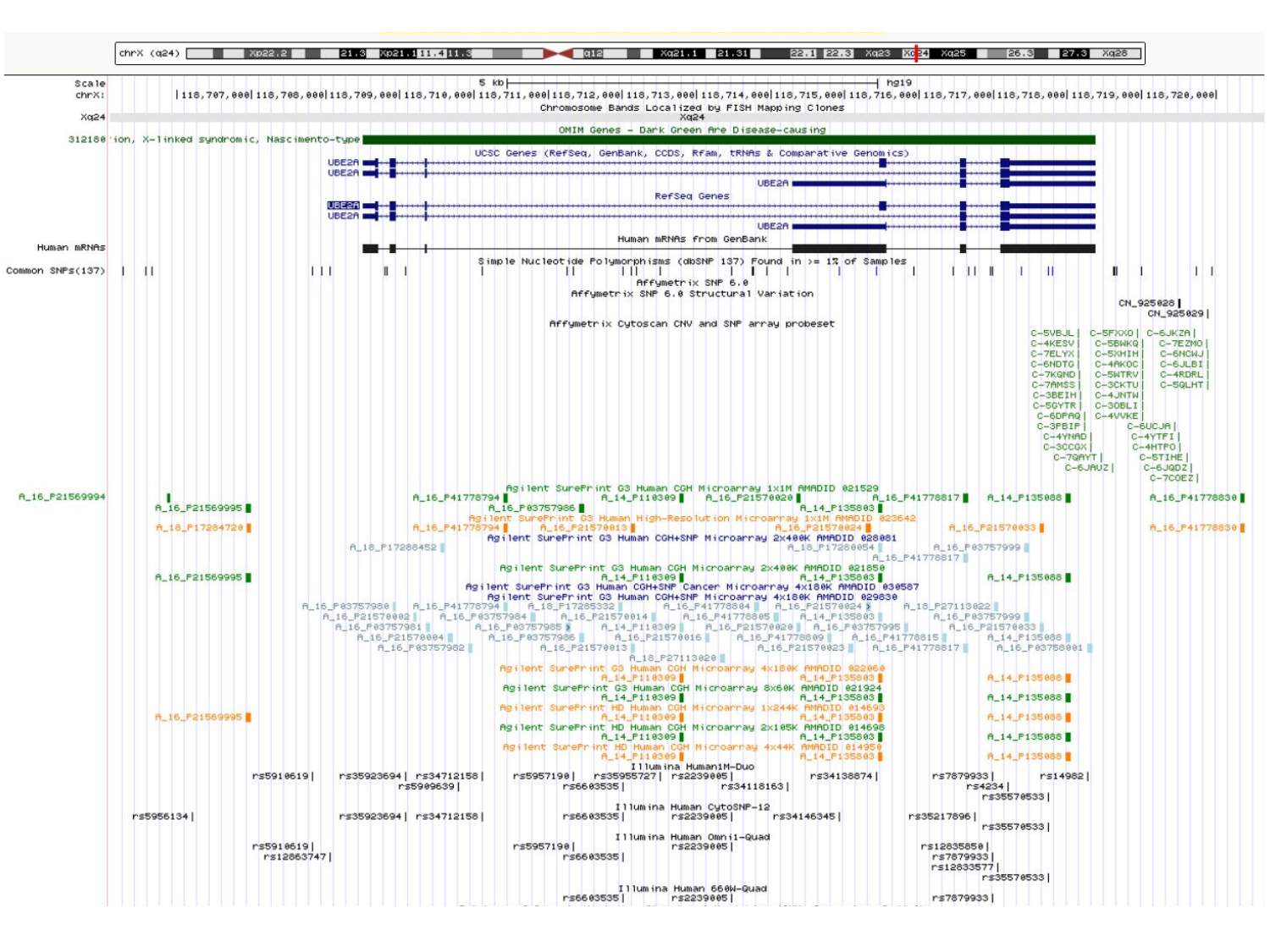

#

Supplement: Additional file 3: Figure S3 — Coverage of the UBE2A region with array markers – comparison of different commercially available array types (according to the UCSC browser, hg19, as of June 2013). Not all array types sufficiently cover the UBE2A region. Two arrays depicted here (Affymetrix SNP 6.0 and Affymetrix Cytoscan HD array) contain no markers at all or only markers in the last exon so that intragenic deletions as in family 1 would have been missed. [file 1750-1172-8-146-S3.pptx]
